# Usability of an Automated System for Real-Time Monitoring of Shared Decision-Making for Surgery: Mixed Methods Evaluation

**DOI:** 10.2196/46698

**Published:** 2024-04-10

**Authors:** Christin Hoffmann, Kerry Avery, Rhiannon Macefield, Tadeáš Dvořák, Val Snelgrove, Jane Blazeby, Della Hopkins, Shireen Hickey, Ben Gibbison, Leila Rooshenas, Adam Williams, Jonathan Aning, Hilary L Bekker, Angus GK McNair, The ALPACA Study Team

**Affiliations:** 1 National Institute for Health and Care Research Bristol Biomedical Research Centre, Bristol Centre for Surgical Research Bristol Medical School: Population Health Sciences University of Bristol Bristol United Kingdom; 2 Patient representative Bristol United Kingdom; 3 North Bristol NHS Trust Bristol United Kingdom; 4 Improvement Academy, Bradford Royal Infirmary Bradford Teaching Hospitals NHS Foundation Trust Bradford United Kingdom; 5 University Hospitals Bristol and Weston NHS Foundation Trust Bristol United Kingdom; 6 Leeds Unit of Complex Intervention Development (LUCID) Leeds Institute of Health Sciences, School of Medicine University of Leeds Leeds United Kingdom; 7 The Research Centre for Patient Involvement (ResCenPI) Department of Public Health Aarhus University Central Denmark Region Denmark

**Keywords:** surgery, shared decision-making, patient participation, mixed methods, surgery, real-time measurement, patient-reported measure, electronic data collection, usability, data collection, patient reported, satisfaction, mobile phone

## Abstract

**Background:**

Improving shared decision-making (SDM) for patients has become a health policy priority in many countries. Achieving high-quality SDM is particularly important for approximately 313 million surgical treatment decisions patients make globally every year. Large-scale monitoring of surgical patients’ experience of SDM in real time is needed to identify the failings of SDM before surgery is performed. We developed a novel approach to automating real-time data collection using an electronic measurement system to address this. Examining usability will facilitate its optimization and wider implementation to inform interventions aimed at improving SDM.

**Objective:**

This study examined the usability of an electronic real-time measurement system to monitor surgical patients’ experience of SDM. We aimed to evaluate the metrics and indicators relevant to system effectiveness, system efficiency, and user satisfaction.

**Methods:**

We performed a mixed methods usability evaluation using multiple participant cohorts. The measurement system was implemented in a large UK hospital to measure patients’ experience of SDM electronically before surgery using 2 validated measures (CollaboRATE and SDM-Q-9). Quantitative data (collected between April 1 and December 31, 2021) provided measurement system metrics to assess system effectiveness and efficiency. We included adult patients booked for urgent and elective surgery across 7 specialties and excluded patients without the capacity to consent for medical procedures, those without access to an internet-enabled device, and those undergoing emergency or endoscopic procedures. Additional groups of service users (group 1: public members who had not engaged with the system; group 2: a subset of patients who completed the measurement system) completed user-testing sessions and semistructured interviews to assess system effectiveness and user satisfaction. We conducted quantitative data analysis using descriptive statistics and calculated the task completion rate and survey response rate (system effectiveness) as well as the task completion time, task efficiency, and relative efficiency (system efficiency). Qualitative thematic analysis identified indicators of and barriers to good usability (user satisfaction).

**Results:**

A total of 2254 completed surveys were returned to the measurement system. A total of 25 service users (group 1: n=9; group 2: n=16) participated in user-testing sessions and interviews. The task completion rate was high (169/171, 98.8%) and the survey response rate was good (2254/5794, 38.9%). The median task completion time was 3 (IQR 2-13) minutes, suggesting good system efficiency and effectiveness. The qualitative findings emphasized good user satisfaction. The identified themes suggested that the measurement system is acceptable, easy to use, and easy to access. Service users identified potential barriers and solutions to acceptability and ease of access.

**Conclusions:**

A mixed methods evaluation of an electronic measurement system for automated, real-time monitoring of patients’ experience of SDM showed that usability among patients was high. Future pilot work will optimize the system for wider implementation to ultimately inform intervention development to improve SDM.

**International Registered Report Identifier (IRRID):**

RR2-10.1136/bmjopen-2023-079155

## Introduction

### Background

Contemporary health care puts patient-centered care at the heart of its delivery [1-4]. Shared decision-making (SDM) is a form of communication that promotes a dialogue between those involved in making health care choices. Therefore, treatment decisions are based on a shared understanding between patients and health care professionals of the evidence base for treatment and prognosis, patient values, preferences and beliefs, and clinical reasoning to personalize service delivery [5]. SDM is desired by patients and has become a key priority for health care systems globally [6-9]. Ensuring high-quality SDM when discussing and deciding treatments with patients can have many benefits, such as reduced information asymmetry or health service use [10,11]. It has been shown to contribute to good patient outcomes and satisfaction [12-15].

Globally, approximately 310 million operations are performed annually [16]. Surgery is often the only available treatment for a wide variety of minor and major medical conditions, and people increasingly choose surgical treatment (5.3% increase from 2009 to 2014 in the United Kingdom) [17]. Improving surgical patients’ experience of SDM before surgery is particularly important because the effects of surgery are immediate and nonreversible. Patients cannot decide to discontinue treatment if the benefits fall short of expectations or side effects become unacceptable. Furthermore, making good surgical decisions may avoid negative impacts on health service costs (eg, through canceled operations) and patient outcomes [18-20].

Strategies aimed at improving SDM in complex health care settings can range from communication skills workshops for health care professionals [21] to educational videos [22] and booklets for patients [23]. However, their effects are mixed [14,15]. Systematic reviews of evidence to improve SDM conclude that achieving long-term change is likely to necessitate interventions that support the implementation of strategies at the organization, clinician, and patient levels [24-26]. However, there is uncertainty about how to realize change on a large scale across health care systems [27-33]. One recommended way to achieve this is through routine monitoring of patients’ experience of SDM [34], but robust methods are lacking. Existing approaches to data collection are delayed, potentially affecting patients’ accounts of their experience and impacting the ability to respond quickly and effectively before surgical treatments. Advances in technology mean that novel approaches to assessing patients’ experiences of SDM can incorporate automated, electronic data capture close to the point of treatment consultations. This offers opportunities for providing information more accurately and in a timely manner, offering an effective way to develop interventions to improve SDM before surgery. Systems routinely collecting electronic patient-reported measure (ePRM) data in other contexts have been shown to improve care and outcomes for patients, including quality of life outcomes in pediatric dermatology [35] or symptom reporting in chronic kidney disease [36]. We developed a novel system to routinely monitor patients’ experience of SDM automatically and in real time.

The evaluation of existing ePRM systems highlights the importance of user-friendly processes for their optimal performance [37-40]. Furthermore, the principles of good usability are important because they can be vital to the widespread uptake of ePRM systems by patients and their successful implementation in clinical practice [41-43]. Usability is an outcome defined as the extent to which the system can be used by specified users [44]. Several methods are available to evaluate measures of usability in health care [45-49]. A widely used framework contains standards set by the International Organization for Standardization (ISO) [50,51]. The guidelines recommend evaluating and optimizing the concepts of system effectiveness (the ability of participants to complete the survey), system efficiency (resources required to complete the questionnaire), and user satisfaction (subjective opinions of participants’ experience with the measurement system) to achieve good usability.

### Aim and Objectives

We aimed to examine the usability of a novel, automated, real-time measurement system to monitor surgical patients’ experience of SDM. The specific objectives were to evaluate the measurement system’s (1) effectiveness, (2) efficiency, and (3) user satisfaction among a large sample of surgical patients from a wide range of surgical specialties.

## Methods

We used quantitative and qualitative methods to examine usability by evaluating the indicators and metrics related to system effectiveness, system efficiency, and user satisfaction. This study adhered to the ISO guideline 9241-11:2018 and followed recommendations for the usability testing of electronic patient-reported outcome measures [49,51].

### Context and Setting

This study is part of a wider project to develop, pilot, and evaluate a decision-support intervention that uses real-time monitoring of patients’ experiences to improve SDM (the ALPACA Study [52]). The project was initially set up as a quality improvement project at a large acute National Health Service (NHS) Trust in England, United Kingdom, which provides a range of acute and specialized clinical care services in South West England.

To facilitate automated, real-time data collection of patients’ experience of SDM, a customizable off-the-shelf ePRM system (Cemplicity) was procured from a third-party software provider in March 2021. The software provider is an ISO 2001 certified, NHS-authorized ePRM provider, compliant with necessary accessibility and health data governance standards (eg, General Data Protection Regulations and Digital Technology Assessment Criteria). Before deployment and customization, the software provider tested the system development and design. Specifically, the prior rollout of the software across 6 countries and over 3000 health care institutions incorporated feedback from users across different health care settings and patients of diverse age groups, technology literacy, and health confidence. All measurement system interfaces are mobile optimized.

Customization for the purpose of this study was undertaken in collaboration with the software provider and included adapting the following: (1) the system’s content and layout to include instruments to assess patients’ experience of SDM and (2) data capture mechanisms to implement the system in the NHS Trust.

To assess patients’ experience of SDM, 2 validated and widely used patient-reported measures were selected to measure SDM (CollaboRATE and SDM-Q-9). These were chosen by consensus within the study team, which was informed by a systematic review of SDM measurement instruments [53], national guidelines [25], and recommendations and use within the NHS clinical practice [34,54,55]. CollaboRATE is a 3-item instrument measured on a 10-point scale with answer options ranging from 0 (“no effort was made”) to 9 (“every effort was made”). SDM-Q-9 consists of 9 items measured on a 6-point scale with answer options ranging from “completely disagree” to “completely agree.” The measurement properties of both instruments have been demonstrated to be acceptable [56,57]. The measurement instruments were operationalized into a 12-question electronic survey format, branded to match the NHS Trust guidelines, and integrated into the patient-facing measurement system. Screenshots of the customized content are presented in Multimedia Appendix 1.

**Figure 1:**
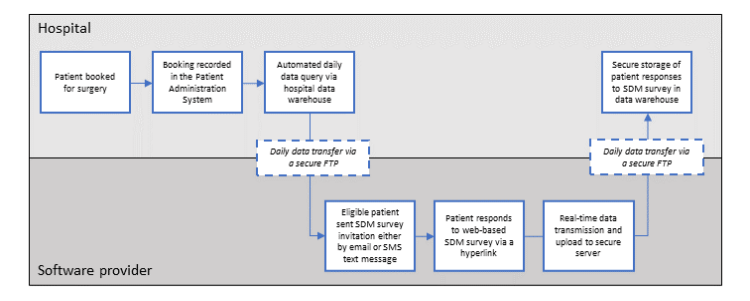
Flow diagram of the process of automated real-time shared decision-making (SDM) monitoring through the measurement system. FTP: file transfer protocol.

To implement the measurement system, secure data exchange processes were established between the software provider and the NHS Trust’s information technology system and subsequently widened to various patient cohorts within the surgical departments. Specifically, SQL data queries were developed to identify and extract details of patients booked for surgery from the electronic patient record system that routinely records the patients’ demographic and clinical information. The queries were designed to run automatically, securely transferring data from the hospital to the software provider on a daily basis. The 2 SDM measures were administered to patients upon being booked for surgery, with invitations sent either by email or SMS text messaging if no email address was available. Patient responses were received and processed using the measurement system. A reciprocal data feed securely returned response data to the hospital data warehouse for secure storage. A flow diagram of the measurement system process is provided in Figure 1.

### Study Steering Group

A multidisciplinary study steering group was convened and consisted of a patient and public contributor, health care professionals, methodologists, social scientists, statisticians, and health services researchers. Regular meetings ensured the group’s strategic oversight throughout and sought their input into the study design, research activities, and analyzing and interpreting results.

### Patient and Public Involvement

We invited a patient and public contributor with lived experience of surgery to the study steering group, which was set up as part of the wider project. The input was sought from the patient and public contributor as appropriate throughout the study (eg, review of patient-facing materials, including survey invitation and instructions, and interim findings from qualitative analyses). In addition, we organized a patient and public advisory meeting which 6 public contributors attended for 1 hour via a Zoom (Zoom Video Communications, Inc) meeting. The aim of the meeting was to obtain patient and public perspectives on the overall project plan and its key challenges. The topics discussed included recruitment, acceptability, and satisfaction with the measurement system, which informed the design aspects of this study.

### Usability Concepts

The usability of the measurement system was examined by evaluating metrics and indicators relevant to 3 concepts, including system effectiveness, system efficiency, and user satisfaction. The definitions are summarized in Textbox 1, and the details of their assessment are described subsequently.

**Definitions of usability concepts.**

- System effectiveness: the ability of participants to perform tasks to achieve predetermined goals completely and accurately, without negative consequences (eg, poor layout of the system interface leading to participants missing or accidentally selecting system options) [36,49-51].
- System efficiency: the amount of participant resources required to achieve the prespecified goals [49,58].
- User satisfaction: the subjective opinions of the participants based on their experience of interacting with the system [49]. This includes any subjective reports about likes, dislikes, and recommendations for changes [51].

### Participants and Procedures

**Figure 2:**
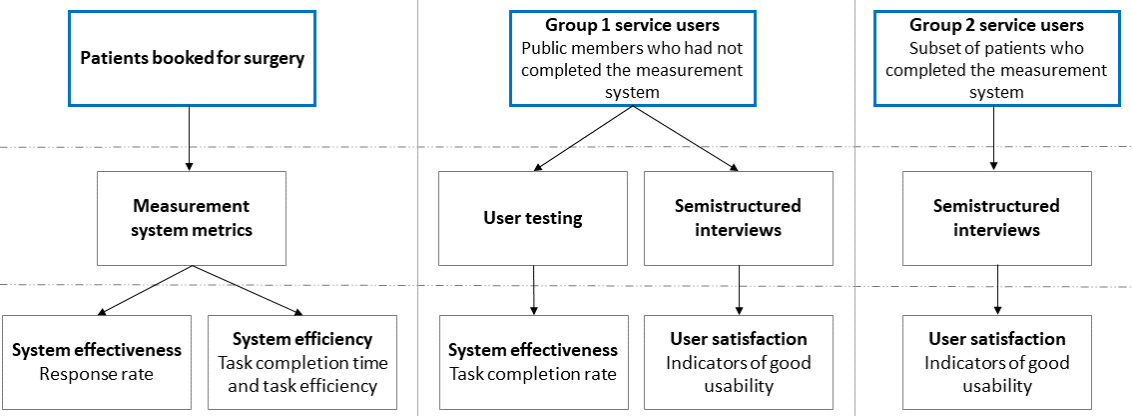
Overview of participant cohorts, data collection procedures, and usability concepts.

We used multiple cohorts of participants and procedures for quantitative and qualitative data collection for this study. Figure 2 illustrates the different cohorts of participants and provides an overview of the data collection procedures used to evaluate the usability concepts.

### Participants and Recruitment

To obtain quantitative measurement system metrics to assess system effectiveness and efficiency (refer to the Quantitative Analysis section for further details), automated, real-time data collection was conducted between April 1 and December 31, 2021, and rolled out across 7 surgical departments: orthopedic, urology, gynecology, neurosurgery, gastrointestinal, vascular and breast. We included adult patients booked for elective surgery in these 7 specialties. Patients aged <18 years, those without the capacity to consent for medical procedures, those undergoing emergency and endoscopic procedures, and those without access to an appropriate internet-enabled device (ie, mobile phone, smartphone, PC, tablet, or similar device) were excluded.

We recruited 2 further groups of service users for user testing and interviews to obtain quantitative and qualitative data to assess system effectiveness and user satisfaction.

Group 1 participants were individuals who had not engaged with the measurement system before user-testing sessions to ensure naive user interactions [59] (refer to the User Testing section for detailed user-testing methods). Service users with experience of surgery were recruited through patient experience panels within 2 NHS Trusts (North Bristol NHS Trust and Bradford Teaching Hospitals NHS Foundation Trust). A panel coordinator identified and approached potential participants via email containing a recruitment advertisement. Sampling was purposive to achieve the maximum possible variation in recognized protected characteristics (eg, sex, disability, and race) and experience of surgery.

Group 2 participants were individuals who had engaged with the measurement system to explore user satisfaction after interacting with the system (refer to the *Semistructured Interviews* section for more details). These were a subset of eligible patients who completed the measurement system. A member of staff with authorized access to the patient administration system and patient response data stored in the data warehouse recruited participants via telephone. We used a purposive sampling strategy to achieve variation in characteristics, including age, ethnicity, sex, type of surgery received, and experience of good or bad SDM (identified through survey responses).

### Procedures

#### Measurement System Metrics

Relevant metrics automatically collected by the measurement system were used to examine usability quantitatively (eg, responses to questionnaire items and timestamps for starting and submitting the survey). Unique entries were recorded for each patient who received the invitation to complete the measurement system. Entries and corresponding data collected between April 1 and December 31, 2021, were available for analysis.

#### User Testing

Postdeployment user-testing sessions were conducted between June and December 2021 and were performed in a simulated environment.

Group 1 participants were invited to participate in a one-to-one 1-hour videoconference via Zoom with a researcher to complete the measurement system live. Sessions began by reminding participants about the aim and the process of user testing the measurement system. Service users were then sent an SMS text message or email invitation (depending on their preference) that included a test link to the survey. Specific user-testing links were set up to allow simulated completion of the measurement system (ie, responses were not used for live response data). Sessions assessed system effectiveness (including any issues related to system functionality or completion). A concurrent think-aloud technique was applied to vocalize reactions and thinking processes [60-62], supplemented with observational notes of any difficulties encountered [63,64].

User-testing sessions were conducted by 1 member of the study team who had experience in think-aloud methods (AGKM or CH). A topic guide was developed to guide conversations (Multimedia Appendix 2). Sessions were audio recorded and transcribed using unique identifiers to ensure anonymity. Field notes and any problems during the measurement system completion were recorded in a table using Excel (Microsoft Corp).

#### Semistructured Interviews

We conducted semistructured, in-depth interviews using retrospective probing to explore the service users’ views about the indicators of usability of the measurement system [65].

Interviews were conducted with group 1 participants following user testing via the same web-based videoconferencing software. Group 2 participants were invited to take part in an approximately 30- to 45-minute phone or videoconferencing call (according to their preference) during which they reflected on completing the measurement system. The conversations followed a previously tested and refined topic guide that was based on standard usability concepts [51]. An example topic guide can be found in Multimedia Appendix 2.

Interviews were performed by either of the 2 researchers (AGKM or CH), audio recorded, and anonymized during transcription.

### Analysis

#### Quantitative Analyses

All quantitative analyses were performed by 3 researchers (TD, AGKM, and CH) using the statistical software package STATA (version 16.0; StataCorp LLC).

##### System Effectiveness

We assessed system effectiveness by calculating the user task completion rate based on usability testing sessions and the survey response rate based on measurement system metrics [66].

The user task completion rates were calculated as a percentage of tasks completed by the total number of tasks. A process map was created defining the number and type of tasks (or steps) required to complete the measurement system. Successful completion means that all tasks were completed without user errors. User errors were deviations or problems encountered that interfered with successful task completion. Noncritical errors were defined as those that were successfully addressed by the testers themselves following instructions from the observer. Critical errors were those that required the observer to intervene or take remedial actions.

The survey response rate was calculated as a percentage (number of completed surveys/number of patients invited × 100). Surveys were considered complete when responses to all 3 items of the CollaboRATE measure and at least 7 out of 9 items of the SDM-Q-9 measure were returned.

##### System Efficiency

We assessed system efficiency by calculating the task completion time and task efficiency based on measurement system metrics [58,66].

The task completion time was defined as the time participants took from the first activity (starting the survey by following the hyperlink) to the last activity (submission of the survey). Task efficiency was defined as the time spent to complete each task (timestamps were recorded in the following format: hh:mm:ss). Analyses were based on those who completed the measurement system for whom typical first and last activity timestamps were available (ie, atypical timestamps were those with no recorded activity time). Extreme outliers were excluded because the system allowed service users to leave and later return to the survey and continue submission (eg, the next day or the following week). These were defined as those with the task completion time >3 times the IQR [67].

#### Qualitative Analyses of User Satisfaction

User satisfaction was assessed by evaluating service users’ self-reported experiences of using the system through user-testing sessions and semistructured interviews [66]. Discussions explored perceptions of usability aspects, including service users’ interpretation of the system’s ease of use and navigation, their satisfaction with instructions and visual display, and the likelihood of using the system again or recommending it to others.

Transcripts obtained from user-testing sessions and semistructured interviews were uploaded to the qualitative data management software NVivo (version 20.5.1; QRS International) and analyzed using thematic analysis [68]. This involved systematic coding of data to identify commonly mentioned concepts within the data and to develop themes and subthemes. The coding was inductive and iterative and followed predefined steps of data familiarization, generation of initial codes, and searching for themes. Coding was performed by 2 researchers independently who met regularly to review themes. Analysis and interpretation of qualitative data were further supported in 2 ways. First, a report of the interim findings was produced and discussed with the wider multidisciplinary steering group. Second, the presentation of interim findings to the patient and public advisory group sought further input.

### Ethical Considerations

This study was part of a project spanning quality improvement and research. Therefore, it was subject to 2 governance processes requiring separate approvals. Monitoring patients’ experience of SDM in routine clinical practice was initially approved through a quality improvement proposal at North Bristol NHS Trust (reference: Q80008). This was then incorporated into a larger program of work, where all processes were approved through the appropriate governance framework (Consent and SDM Program Board, reporting to the Clinical Effectiveness and Audit Committee). Ethics approval for conducting interviews with NHS patients was granted by the NHS Health Research Authority North West – Liverpool Central Research Ethics Committee (reference: 21/PR/0345). Participants provided electronic consent through a link to a secure data management platform (version 11.1.18, REDCap [Research Electronic Data Capture]; Vanderbilt University) [69] before any study activity commenced.

## Results

### Participants and Procedures

A total of 5794 surgical patients received invitations to complete the survey and for whom unique entries were recorded in the measurement system. Of these, 2254 returned the completed surveys (refer to Table 1 for patient characteristics) and provided data for the analysis of measurement metrics.

**Table 1:** Characteristics of patients who completed the measurement system (N=2254).

Characteristics		Patients, n (%)
**Sex**		
	Female	1243 (55.15)
	Male	1011 (44.85)
**Age group (y)**		
	<29	170 (7.54)
	30 to 39	213 (9.45)
	40 to 49	277 (12.29)
	50 to 59	529 (23.47)
	60 to 69	555 (24.62)
	70 to 79	402 (17.83)
	≥80	108 (4.79)
**Ethnicity^a^**		
	Other	104 (4.61)
	White British	977 (43.35)
**Specialty**		
	Breast	278 (12.33)
	Colorectal	67 (2.97)
	General	194 (8.61)
	Gynecology	106 (4.7)
	Neuro	288 (12.78)
	Trauma, orthopedics and spinal	555 (24.62)
	Upper gastrointestinal	41 (1.82)
	Urology	584 (25.91)
	Vascular	141 (6.26)

^a^Missing data: n=1173.

A total of 25 service users (group 1: n=9; group 2: n=16) participated in user-testing sessions and semistructured interviews.

In group 1, a total of 9 service users completed 8 user-testing sessions. Most sessions were completed on a one-to-one basis (7/9, 78%). One session was completed with 2 participants, which included 1 service user with disability and their caregiver who provided additional support. All sessions were held via videoconference and lasted for an average duration of 43 (SD 15.1; range 29-78) minutes. Service users in this group were mostly female participants (6/9, 67%) and self-identified as Asian (1/9, 11%), other White background (1/9, 11%), and White British (7/9, 78%). Details about the surgical experience were known for 4 service users who represented orthopedic (2/4, 50%), upper gastrointestinal (1/4, 25%), and ophthalmic (1/4, 25%) specialties.

In group 2, 16 service users completed semistructured interviews between June and November 2021. Most interviews were conducted via telephone (15/16, 94%), with 1 (6%) interview conducted via videoconference, lasting for an average duration of 36 (SD 9.9; range 21-50) minutes. Most service users in group 2 were female participants (10/16, 62%) and were 51 (SD 15.8) years on average. All participants were from a White British background (16/16, 100%). Efforts were made to recruit participants from a wide range of ethnic minority backgrounds; however, due to a large amount of missing data (Table 1), this was unsuccessful. The characteristics of group 2 participants are presented in Table 2.

**Table 2:** Characteristics of group 2 service users (n=16).

Characteristics		Service users, n (%)
Age (y), mean (SD; range)		51 (15.8; 23-80)
**Sex, n (%)**		
	Female	10 (62)
	Male	6 (38)
**Ethnicity, n (%)**		
	White British	16 (100)
**Surgery type, n (%)**		
	Breast	3 (19)
	Colorectal	2 (13)
	General	2 (13)
	Gynecology	1 (6)
	Trauma and orthopedics	2 (13)
	Urology	5 (31)
	Vascular	1 (6)

### Usability Concepts

#### System Effectiveness

A process map to assess task completion contained 19 tasks (or steps) required to complete the measurement system. Tasks ranged from “Open text message/email” to “Click on ‘Submit’” and are detailed in Multimedia Appendix 3.

A total of 171 tasks across 8 user-testing sessions were submitted by all 9 group 1 participants. One service user reported 2 noncritical errors across 2 tasks when completing the measurement system using a mobile phone. The first error occurred following task 1 “Open text message.” This forced an additional step to resolve a pop-up notification which prompted the service user to select an internet browser to open the survey link. The second error occurred following task 5 “Select response to question 1.” The displayed answer options for CollaboRATE item 1 were cut off at 8, not presenting answer option 9 (every effort was made). Further scrolling was required by the service user to be able to select the answer option 9. Both noncritical errors were managed and resolved without requiring observer input. Consequently, a total completion rate of 98.8% (169/171) was achieved. No critical errors or failures in completing the tasks were reported.

The survey response rate was 38.9% (2254 completed surveys/5794 patients invited × 100).

#### System Efficiency

Out of the 2254 responses available, 1106 (49.07%) were excluded from analysis. These 1106 responses included 719 (65.01%) responses with an atypical timestamp (ie, no activity time was recorded because the timestamp for the first and last activity was 00:00:00, which was identified as a technical issue and rectified by the software provider) and 387 (34.99%) responses identified as extreme outliers (ie, the task completion time was >12 min). Assessment of the completion time of 1148 (50.93%) of the 2254 responses showed that service users required an average median duration of 3 (IQR 2-4) minutes to complete the measurement system. Calculations of task efficiency showed that the average median time taken per task was 9 (IQR 6-13) seconds.

#### User Satisfaction

Analysis of qualitative data from user-testing sessions and semistructured interviews with a subset of patients revealed four main themes related to user satisfaction as follows: (1) acceptability, (2) ease of access to the system, (3) ease of use, and (4) satisfaction with the measurement system.

##### Acceptability

###### Indicators of Good Acceptability

Service users who were interviewed as part of the qualitative data collection frequently commented on the low burden of completing the measurement system, suggesting good acceptability among the participants. This was mainly because of the low number of questions contributing to the measurement system being considered quick and straightforward to use:

Short survey, key thing—not too much of your time.

PT9, group 1

I did it from my phone so yes it was very straightforward.

PT13, group 2

I don’t remember feeling any burden [...], it was quite easy.

PT19, group 2

I don’t think it seemed too long. It was enough. To be honest, if it had been a lot more, I probably wouldn’t bother to do it.

PT21, group 2

Furthermore, service users highlighted the common use of web-based surveys to obtain feedback in health care and other general settings. Therefore, they felt a certain level of familiarity with the measurement system, which contributed to the good acceptability:

I thought it was, I mean, pretty standard, you know, arial buttons, nought to ten on how much you disagree, agree, disagree to something so yes, familiar with many other surveys that I’ve seen before.

PT12, group 2

###### Potential Barriers to Acceptability

Some barriers to completing the measurement system were highlighted. For example, participants mentioned that the service users may easily ignore or forget to complete the measurement system as follows:

It’s easy not to [complete the measurement system], I've had them from places, not about health or anything important like that, but it’s easy just [to] think, “Oh, I’ll do that later,” and then never go back to it.

PT7, group 1

Another example included concerns about the number of SMS text messages and surveys received from other sources and the cumulative burden:

I mean the good thing about it is it’s simple and easy and you just get the nudge, but on the other hand there are lots of other nudges coming through at you.

PT19, group 2

This contributed to a small number of service users questioning the credibility of the invitation to complete the measurement system:

Something came through via email which to be honest I wasn’t sure if it was a genuine thing or if it was something else.

PT10, group 2

###### Solutions to Address Barriers

Service users were asked about the usability of solutions to address these issues and included support for reminder emails:

One follow-up is a good idea but not more than one possibly because then people start to feel a bit harassed, but I think a second one is a good idea because of the forgetting thing and they go oh yeah, I’ll do it this time.

PT4, group 1

Service users thought that the use of email would address this problem for some service users:

It is at the top of my email pile again, I’d better do it, so it jogs your memory, texts don’t do that, it’s a very momentary thing, text messaging.

PT13, group 2

Furthermore, service users suggested to increase the personal relevance and awareness of the measurement system:

You’ve got to feel that you’re going to benefit, and it’s really relevant to you, for you to have the interest to do it.

PT7, group 1

They [service users] really, really need to know it’s coming because I don’t know about you but we’re very, very careful what we open and if this just appeared with no warning I wouldn’t open it.

PT4, group 1

Service users mentioned the need to highlight the brevity of the measurement system and the low number of questions:

There are people who will fill them in if they’re told it’s very short, which is why it’s important that it says it’s short.

PT9, group 1

I think sometimes if you open one you can see that it’s 100 questions you just think I probably won’t do that.

PT14, group 2

##### Ease of Access to the System

###### Indicators of Good Ease of Access to the System

All service users were able to access the measurement system without problems and commented on its ease of access through both methods, email and SMS text message:

I think most people nowadays are comfortable with computers and technology.

PT14, group 2

Some service users expressed a preference for using either email or their phone to complete the measurement system. However, there was no conclusive evidence to suggest the superiority of either email or SMS text message:

I guess that for me making it [come to my phone] makes it more accessible ‘cos you don’t have to go in your emails. It automatically comes through and you can do it at any time and reply at any time, so you can do it when it’s convenient to you and its literally just a text on your phone.

PT1, group 1

Although I use a smart phone quite a lot, sometimes it’s difficult to manipulate it, whilst a laptop I find much more easier to use.

PT8, group 1

Furthermore, service users commented on the good comprehensibility and legibility of the content, contributing to good levels of ease of access to the system. For example, comments included that there was a sufficiently large font option for those who required or preferred larger screens:

I think the presentation of it on my phone, and I don’t have a large phone, I just have a small phone, I could read all that quite easily.

PT7, group 1

They were really easy to understand[...] The questions were very clear, I thought they were quite well[...] focused and well explained.

PT24, group 2

###### Potential Barriers to Access to the System

Some service users expressed concerns regarding the system’s ease of access for certain population groups. Most frequently, concerns were raised in connection with older adults and lack of access to technology. Furthermore, considerations included the ease of access to the measurement system for non–English-speaking service users and those with disabilities:

There’s also a certain cohort would be using online. [...] I do think people will miss out but if it’s just being pinged… whether it’s on text or email

PT3, group 1

People that English isn’t their first language, that could be a bit of a consideration.

PT5, group 1

###### Solutions to Address Barriers

The most frequently mentioned solutions were common alternatives to electronic data collection in connection with support measures for questionnaire completion:

I mean there’s probably still a gap with the older generation who wouldn’t be comfortable doing it, and would prefer doing it via communication of phone or in written format.

PT14, group 2

###### Ease of Use

Most often, the simplicity of the system was highlighted in connection with the ease of completing the measurement system. Furthermore, the ease of use was often attributed to the brevity of the measurement system:

I actually thought it was quite simple and quite straightforward and easy.

PT3, group 1

That has been perfectly straightforward, for someone who’s not very IT literate, that was all fine.

PT7, group 1

Yeah, that was very easy, it didn’t take very long [...] I remember it did seem simple

PT23, group 2

Moreover, most service users commented on the visual display, which was perceived as appealing and very clear. The clear layout of the survey contributed to high comprehensibility among participants:

It is very clear and also I quite like the bold type. [...] very clear again and very easy to read.

PT3, group 1

It’s pretty obvious straight off of that where the survey has come from including the logo and almost like the colours of the survey match with the NHS logo [...] I think that part of it makes it really easy.

PT21, group 2

Yeah, that’s laid out really spaced out and easy to read.

PT6, group 1

Service users frequently mentioned the ease of navigation and thought it was “basic and straightforward” (PT20, group 2). Others mentioned further details regarding what they liked about the navigation:

There is no need to zoom in or zoom out or move around a page or click buttons to find the survey so I think all of that aspect is really easy. [...] It’s easy to use and the agree or disagree buttons are really straight to the point.

PT21, group 2

One service user also commented on the loading speed of the survey page:

I think it’s easy to use because it doesn’t take long to load which I think is important.

PT20, group 2

No service user raised concerns that could be considered barriers to the ease of use of the measurement system.

###### Overall Satisfaction With the Measurement System

All service users provided positive feedback regarding the abovementioned themes of acceptability, ease of access to the system, and ease of use, which indicated high satisfaction with the measurement system. General supportive comments were made throughout the user-testing sessions and semistructured interviews:

Yeah, absolutely brilliant. I’ll give that 11 out of 10. [...] Somebody who designed this did a good job.

PT5, group 1

All respondents agreed when asked whether they are likely to complete the measurement system again:

Yeah, I would definitely respond to it again.

PT6, group 1

In addition, there were unprompted comments related to satisfaction with particular features. For example, service users pointed out that they particularly liked the “back buttons” to return to previous questions, the option to pause the measurement system and return at a different time, and the fact that there are contact details of the hospital in case this survey was received in error:

You've got the option, you can go back and change something, or if there was something you were worried about that you’ve done, it’s clear that you can go back.

PT7, group 1

## Discussion

### Principal Findings

This study examined the usability of a novel automated and real-time ePRM system to monitor patients’ experience of SDM in routine clinical practice. We used a large sample from a diverse range of surgical specialties to evaluate system effectiveness, system efficiency, and user satisfaction.

Overall, the evaluation of the measurement system demonstrated good usability. Metrics relevant to the effectiveness and efficiency showed that the system can be used without problems and completed quickly. The results from qualitative testing sessions and interviews with 25 service users showed that the measurement system has good user satisfaction. It was perceived as acceptable, easy to access, and easy to use. Service users identified potential barriers to acceptability and ease of access to the system, which can inform strategies for the optimization of the measurement system.

### Limitations

This study has certain methodological limitations. First, we purposively selected participants to include individuals from a wide socioeconomic background with varying computer literacy skills. While this study exceeded the recommended sample size for usability testing [70-72], service users in our sample were primarily White British (23/25, 92%), English-speaking adults with capacity to consent for medical treatments, and from specific geographic areas of the United Kingdom (West, South West, and North East England). This may limit the generalizability of the study findings. It is uncertain whether the inclusion of more participants from more diverse backgrounds would have elicited different perspectives on the measurement system. Second, only patients who had completed the measurement system were eligible to participate in semistructured interviews. Data protection regulations limited our ability to recruit individuals who had not completed the survey. Therefore, we were unable to explore whether nonengagement with the system was due to reasons related to usability not mentioned by the study participants. Barriers to engagement may align with the themes identified during semistructured interviews, which are partly addressed by ongoing work (refer to the following section). Separately, there is ongoing work which includes conducting follow-up phone calls with patients to explore the reasons for nonengagement. Third, usability may also be evaluated using validated measurement instruments to capture quantitative measures of individuals’ perception of usability from a larger, representative sample size [73,74]. This study did not include such measures in addition to the ePRM to avoid distorting usability outcomes. For example, the additional length of the survey may have affected system efficiency and impacted perceptions of ease of use. Instead, we included a range of methods to assess usability to triangulate the data sources [75].

### Comparison With Prior Work

Existing research has investigated optimal strategies and methods for collecting ePRMs [40,76-79]. The usability evaluation of electronic platforms is common and has been fundamental in optimizing systems to collect ePRMs across a range of health care settings [80] and also within surgery [81,82]. Less is known about systems that monitor patients’ experiences automatically and in real time. We are aware of only 1 recently published protocol describing a similar measurement system [83], but we were unable to identify studies with specific relevance to surgery or SDM. Our study addresses this gap and provides insights into the usability of an automated measurement system that monitors ePRMs for SDM in real time. The measurement system in our study was evaluated for service users undergoing surgical treatment; however, the findings may be applicable to other health care settings.

Evidence of good usability of an automated measurement system that captures surgical patients’ experiences in real time supports the measurement systems’ potential for scalability. The use of the system is recommended in similar health care settings where policy makers or official bodies wish to audit or monitor patients’ experiences of SDM or aim to inform interventions to improve SDM before treatment. System effectiveness and efficiency are central components to service users’ successful interaction with any system [51]. The usability concepts evaluated have been shown to be key in other systems rolled out in surgical departments [84] and are likely to play a role in the wider adoption of the measurement system [85]. This study showed that service users were able to successfully complete the measurement system and that they required little time and effort to do so. In addition, good user satisfaction is vital to a system’s sustainability and is used as a measure of the success of digital information systems within health care organizations worldwide [86-88]. User satisfaction with ePRM systems and perceived acceptability, in particular, have been shown to be key to their uptake among stakeholders [89,90]. The qualitative evidence obtained from service users in this study demonstrated good acceptability, ease of access to the system, and ease of use, which suggests low concern regarding user satisfaction. Some steps to optimize the system to address identified usability concerns and adapt SDM measurement to other care contexts [91] might be necessary before a wider rollout to other health care settings.

This study highlighted well-known barriers to ease of access to electronic measurement systems [92,93]. Specifically, literacy with electronic systems can be lower in older and frail adults and among individuals without capacity to consent [94-97]. While the measurement system response rate in this study (2254/5794, 38.9%) was notably higher compared to those reported in other studies evaluating measurement systems (eg, 18% in the study by Iversen et al [98], 20% in the study by Bliddal et al [99], or 30% in the study by Arner [82]), it may be indicative of such barriers experienced by surgical patients. The solutions to improve ease of access identified in this study include additional paper-based methods. Furthermore, barriers may be overcome through assisted data collection using a tablet computer at the point of care [100]. Additional resources may be required to ensure full and accurate data capture for adults without capacity to consent to medical treatments completing the measurement system [101]. Similarly, language barriers have been shown to affect service users’ ease of access to the system and the quality of responses to ePRM systems [93,102]. Translating content can be key to addressing such language barriers, as demonstrated by widely used quality of life measures [103]. Further work is currently ongoing to address relevant issues to maximize inclusivity (ISRCTN [International Standard Randomised Controlled Trial Number] registry ID: 17951423). Specifically, this line of work seeks to explore the views of underserved groups (eg, limited income, older age, and ethnic minority groups) using qualitative methods to understand how the use of the system and future intervention development can be optimized to maximize inclusivity. This work will consider nondigital materials, translation of study materials, measurement system content, and measurement instruments using appropriate guidance [104] and will include non-English qualitative data collection. Detailed methods will be reported in a separate publication.

High-quality SDM can be a moderator and mediator of health and care quality [105], addressing the challenges of true patient-centered care (eg, reducing asymmetry in medical knowledge between patients and surgeons and addressing issues of individual preferences). To improve patients’ experiences of SDM before surgery, additional intervention development work is needed to complement automated, real-time monitoring of SDM experiences. Evidence from other clinical settings suggests that interventions, including real-time feedback, in addition to routine monitoring of ePRMs, can lead to improvements in outcomes or clinical performance [81,106-108]. This study demonstrated the good usability of a measurement system that automatically collects, stores, and retrieves ePRM data and is ready to provide feedback on this information in digital format near to real time. This suggests that the system is ready to provide instantaneous feedback on surgical patients’ experience of SDM to clinical teams, which has the potential to improve SDM. Future work will explore the optimal design and feasibility of feedback mechanisms and examine the acceptability of the system. Refinements to optimize the usability and inclusivity of the system are required before evaluating the effectiveness of an intervention to improve SDM. Key to this work will be obtaining wider perspectives from other stakeholders involved in the intervention (eg, health care professionals and stakeholders from the lower-income, ethnic minority, and older age groups). In the long term, strategies to facilitate the implementation of the measurement system in routine clinical care will be investigated and evaluated using evidence-based approaches to intervention design [109].

### Conclusions

We examined the usability of a measurement system for automated and real-time ePRM collection to monitor patients’ experience of SDM in a large sample using 2 brief, validated instruments. The findings suggest good usability and support scalability of the measurement systems to other secondary health care institutions and will inform its optimization. Complementary work is currently exploring the feasibility and acceptability of monitoring and feedback experience of SDM with patient and professional stakeholders. Future implementation and formal evaluation of the measurement system will be performed to establish whether routine monitoring and feedback of patients’ experiences has the potential to improve SDM for surgical patients.

## Appendix

Multimedia Appendix 1 Example screenshots of the measurement system.

Multimedia Appendix 2 Topic guide.

Multimedia Appendix 3 Process map of tasks.

## Abbreviations

ePRM: electronic patient-reported measure
ISO: International Organization for Standardization
ISRCTN: International Standard Randomised Controlled Trial Number
NHS: National Health Service
REDCap: Research Electronic Data Capture
SDM: shared decision-making

## References

[1] (2021). The NHS constitution for England. Department of Health and Social Care.

[2] (2019). The NHS long term plan. National Health Service.

[3] (2013). Good surgical practice. Royal College of Surgeons.

[4] (2020). Decision making and consent - ethical guidance. General Medical Council.

[5] Salzburg Global Seminar (2011). Salzburg statement on shared decision making. BMJ.

[6] Elwyn G, Laitner S, Coulter A, Walker E, Watson P, Thomson R (2010). Implementing shared decision making in the NHS. BMJ.

[7] Briss P, Rimer B, Reilley B, Coates RC, Lee NC, Mullen P, Corso P, Hutchinson AB, Hiatt R, Kerner J, George P, White C, Gandhi N, Saraiya M, Breslow R, Isham G, Teutsch SM, Hinman AR, Lawrence R, Task Force on Community Preventive Services (2004). Promoting informed decisions about cancer screening in communities and healthcare systems. Am J Prev Med.

[8] Goto Y, Miura H (2022). Challenges in promoting shared decision-making: towards a breakthrough in Japan. Z Evid Fortbild Qual Gesundhwes.

[9] Lu C, Li X, Yang K (2019). Trends in shared decision-making studies from 2009 to 2018: a bibliometric analysis. Front Public Health.

[10] Hughes TM, Merath K, Chen Q, Sun S, Palmer E, Idrees JJ, Okunrintemi V, Squires M, Beal EW, Pawlik TM (2018). Association of shared decision-making on patient-reported health outcomes and healthcare utilization. Am J Surg.

[11] Tai-Seale M, Elwyn G, Wilson CJ, Stults C, Dillon EC, Li M, Chuang J, Meehan A, Frosch DL (2016). Enhancing shared decision making through carefully designed interventions that target patient and provider behavior. Health Aff (Millwood).

[12] Kashaf MS, McGill E (2015). Does shared decision making in cancer treatment improve quality of life? A systematic literature review. Med Decis Making.

[13] Stacey D, Bennett CL, Barry MJ, Col NF, Eden KB, Holmes-Rovner M, Llewellyn-Thomas H, Lyddiatt A, Légaré F, Thomson R (2011). Decision aids for people facing health treatment or screening decisions. Cochrane Database Syst Rev.

[14] Durand M, Carpenter L, Dolan H, Bravo P, Mann M, Bunn F, Elwyn G (2014). Do interventions designed to support shared decision-making reduce health inequalities? A systematic review and meta-analysis. PLoS One.

[15] Shay LA, Lafata JE (2015). Where is the evidence? A systematic review of shared decision making and patient outcomes. Med Decis Making.

[16] Weiser TG, Haynes AB, Molina G, Lipsitz SR, Esquivel MM, Uribe-Leitz T, Fu R, Azad T, Chao TE, Berry WR, Gawande AA (2015). Estimate of the global volume of surgery in 2012: an assessment supporting improved health outcomes. Lancet.

[17] Abbott TE, Fowler AJ, Dobbs TD, Harrison EM, Gillies MA, Pearse RM (2017). Frequency of surgical treatment and related hospital procedures in the UK: a national ecological study using hospital episode statistics. Br J Anaesth.

[18] Davison BJ, Matthew A, Gardner AM (2014). Prospective comparison of the impact of robotic-assisted laparoscopic radical prostatectomy versus open radical prostatectomy on health-related quality of life and decision regret. Can Urol Assoc J.

[19] Diefenbach MA, Mohamed NE (2007). Regret of treatment decision and its association with disease-specific quality of life following prostate cancer treatment. Cancer Invest.

[20] Walsh T, Barr PJ, Thompson R, Ozanne E, O'Neill C, Elwyn G (2014). Undetermined impact of patient decision support interventions on healthcare costs and savings: systematic review. BMJ.

[21] Kissane DW, Bylund CL, Banerjee SC, Bialer PA, Levin TT, Maloney EK, D'Agostino TA (2012). Communication skills training for oncology professionals. J Clin Oncol.

[22] Ross L, Ashford AD, Bleechington SJ, Dark T, Erwin DO (2010). Applicability of a video intervention to increase informed decision making for prostate-specific antigen testing. J Natl Med Assoc.

[23] Smith SK, Trevena L, Simpson JM, Barratt A, Nutbeam D, McCaffery KJ (2010). A decision aid to support informed choices about bowel cancer screening among adults with low education: randomised controlled trial. BMJ.

[24] Joseph-Williams N, Lloyd A, Edwards A, Stobbart L, Tomson D, Macphail S, Dodd C, Brain K, Elwyn G, Thomson R (2017). Implementing shared decision making in the NHS: lessons from the MAGIC programme. BMJ.

[25] (2021). Shard decision making NG197. National Institute for Health and Care Excellence.

[26] Légaré F, Adekpedjou R, Stacey D, Turcotte S, Kryworuchko J, Graham ID, Lyddiatt A, Politi MC, Thomson R, Elwyn G, Donner-Banzhoff N (2018). Interventions for increasing the use of shared decision making by healthcare professionals. Cochrane Database Syst Rev.

[27] Ubbink DT, Hageman MG, Legemate DA (2015). Shared decision-making in surgery. Surg Technol Int.

[28] Elwyn G, Scholl I, Tietbohl C, Mann M, Edwards AG, Clay C, Légaré F, van der Weijden T, Lewis CL, Wexler RM, Frosch DL (2013). "Many miles to go …": a systematic review of the implementation of patient decision support interventions into routine clinical practice. BMC Med Inform Decis Mak.

[29] Forcino RC, Meinders MJ, Engel JA, O'Malley AJ, Elwyn G (2020). Routine patient-reported experience measurement of shared decision-making in the USA: a qualitative study of the current state according to frontrunners. BMJ Open.

[30] Scholl I, Hahlweg P, Lindig A, Frerichs W, Zill J, Cords H, Bokemeyer C, Coym A, Schmalfeldt B, Smeets R, Vollkommer T, Witzel I, Härter M, Kriston L (2021). Evaluation of a program for routine implementation of shared decision-making in cancer care: results of a stepped wedge cluster randomized trial. Implement Sci.

[31] Elwyn G, Frosch DL, Kobrin S (2016). Implementing shared decision-making: consider all the consequences. Implement Sci.

[32] Härter M, van der Weijden T, Elwyn G (2011). Policy and practice developments in the implementation of shared decision making: an international perspective. Z Evid Fortbild Qual Gesundhwes.

[33] Zisman-Ilani Y, Chmielowska M, Dixon LB, Ramon S (2021). NICE shared decision making guidelines and mental health: challenges for research, practice and implementation. BJPsych Open.

[34] Shared decision-making: summary guide. NHS England Personalised Care.

[35] Tyack Z, Simons M, McPhail SM, Harvey G, Zappala T, Ware RS, Kimble RM (2021). Improving the patient-centred care of children with life-altering skin conditions using feedback from electronic patient-reported outcome measures: protocol for a hybrid effectiveness-implementation study (PEDS-ePROM). BMJ Open.

[36] Kyte D, Anderson N, Auti R, Aiyegbusi OL, Bishop J, Bissell A, Brettell E, Calvert M, Chadburn M, Cockwell P, Dutton M, Eddington H, Forster E, Hadley G, Ives NJ, Jackson L, O'Brien S, Price G, Sharpe K, Stringer S, Stephenson G, Verdi R, Waters J, Wilcockson A, Williams J (2020). Development of an electronic patient-reported outcome measure (ePROM) system to aid the management of patients with advanced chronic kidney disease. J Patient Rep Outcomes.

[37] van der Krieke L, Emerencia AC, Aiello M, Sytema S (2012). Usability evaluation of a web-based support system for people with a schizophrenia diagnosis. J Med Internet Res.

[38] Richards HS, Blazeby JM, Portal A, Harding R, Reed T, Lander T, Chalmers KA, Carter R, Singhal R, Absolom K, Velikova G, Avery KN (2020). A real-time electronic symptom monitoring system for patients after discharge following surgery: a pilot study in cancer-related surgery. BMC Cancer.

[39] Basch E, Deal AM, Kris MG, Scher HI, Hudis CA, Sabbatini P, Rogak L, Bennett AV, Dueck AC, Atkinson TM, Chou JF, Dulko D, Sit L, Barz A, Novotny P, Fruscione M, Sloan JA, Schrag D (2016). Symptom monitoring with patient-reported outcomes during routine cancer treatment: a randomized controlled trial. J Clin Oncol.

[40] Aiyegbusi OL, Kyte D, Cockwell P, Marshall T, Dutton M, Walmsley-Allen N, Slade A, McMullan C, Calvert M (2019). Patient and clinician perspectives on electronic patient-reported outcome measures in the management of advanced CKD: a qualitative study. Am J Kidney Dis.

[41] van Gemert-Pijnen JE, Nijland N, van Limburg M, Ossebaard HC, Kelders SM, Eysenbach G, Seydel ER (2011). A holistic framework to improve the uptake and impact of eHealth technologies. J Med Internet Res.

[42] O'Cathain A, Croot L, Sworn K, Duncan E, Rousseau N, Turner K, Yardley L, Hoddinott P (2019). Taxonomy of approaches to developing interventions to improve health: a systematic methods overview. Pilot Feasibility Stud.

[43] Yardley L, Morrison L, Bradbury K, Muller I (2015). The person-based approach to intervention development: application to digital health-related behavior change interventions. J Med Internet Res.

[44] Bevan N (1995). Measuring usability as quality of use. Software Qual J.

[45] Maramba I, Chatterjee A, Newman C (2019). Methods of usability testing in the development of eHealth applications: a scoping review. Int J Med Inform.

[46] Dumas JS, Salzman MC (2016). Usability assessment methods. Rev Hum Factors Ergon.

[47] Lin HX, Choong Y, Salvendy G (1997). A proposed index of usability: a method for comparing the relative usability of different software systems. Behav Inf Technol.

[48] Yen PY, Bakken S (2012). Review of health information technology usability study methodologies. J Am Med Inform Assoc.

[49] Aiyegbusi OL (2020). Key methodological considerations for usability testing of electronic patient-reported outcome (ePRO) systems. Qual Life Res.

[50] (2018). ISO 9241-11:2018: ergonomics of human-system interaction: part 11: usability: definitions and concepts. International Organization for Standardization.

[51] Bevan N, Carter J, Earthy J, Geis T, Harker S (2016). New ISO standards for usability, usability reports and usability measures. Proceedings of the 18th International Conference on Human-Computer Interaction. Theory, Design, Development and Practice.

[52] Hoffmann C, Avery KN, Macefield RC, Snelgrove V, Blazeby JM, Hopkins D, Hickey S, Cabral C, Hall J, Gibbison B, Rooshenas L, Williams A, Aning J, Bekker HL, McNair AG, ALPACA Study team (2024). Real-time monitoring and feedback to improve shared decision-making for surgery (the ALPACA Study): protocol for a mixed-methods study to inform co-development of an inclusive intervention. BMJ Open.

[53] Gärtner FR, Bomhof-Roordink H, Smith IP, Scholl I, Stiggelbout AM, Pieterse AH (2018). The quality of instruments to assess the process of shared decision making: a systematic review. PLoS One.

[54] (2019). Personalised care shared decision making summary guide. NHS England and NHS Improvement.

[55] Barr PJ, Forcino RC, Thompson R, Ozanne EM, Arend R, Castaldo MG, O'Malley AJ, Elwyn G (2017). Evaluating CollaboRATE in a clinical setting: analysis of mode effects on scores, response rates and costs of data collection. BMJ Open.

[56] Doherr H, Christalle E, Kriston L, Härter M, Scholl I (2017). Use of the 9-item shared decision making questionnaire (SDM-Q-9 and SDM-Q-Doc) in intervention studies-a systematic review. PLoS One.

[57] Barr PJ, Thompson R, Walsh T, Grande SW, Ozanne EM, Elwyn G (2014). The psychometric properties of CollaboRATE: a fast and frugal patient-reported measure of the shared decision-making process. J Med Internet Res.

[58] Barnum CM, Barnum CM (2011). Establishing the essentials. Usability Testing Essentials: Ready, Set ...Test!.

[59] Hedberg H, Iivari N, Rajanen M, Harjumaa L (2007). Assuring quality and usability in open source software development. Proceedings of the 1st International Workshop on Emerging Trends in FLOSS Research and Development.

[60] van den Haak MJ, de Jong MD, Schellens PJ (2007). Evaluation of an informational web site: three variants of the think-aloud method compared. Tech Commun.

[61] Alshammari T, Alhadreti O, Mayhew PJ (2015). When to ask participants to think aloud: a comparative study of concurrent and retrospective think-aloud methods. Int J Hum Comput Interact.

[62] Khajouei R, Farahani F (2020). A combination of two methods for evaluating the usability of a hospital information system. BMC Med Inform Decis Mak.

[63] Farzanfar R, Finkelstein J, Friedman RH (2004). Testing the usability of two automated home-based patient-management systems. J Med Syst.

[64] Kaufman DR, Patel VL, Hilliman C, Morin PC, Pevzner J, Weinstock RS, Goland R, Shea S, Starren J (2003). Usability in the real world: assessing medical information technologies in patients' homes. J Biomed Inform.

[65] Birns J, Birns JH, Joffre KA, Leclerc JF, Paulsen CA (2002). Getting the Whole Picture: Collecting Usability Data Using Two Methods-Concurrent Think Aloud and Retrospective Probing. https://citeseerx.ist.psu.edu/document?repid=rep1&type=pdf&doi=ba55a9ff388337e7e561fd1db41b3533051746a9.

[66] Geisen E, Romano Bergstrom J, Geisen E, Romano Bergstrom J (2017). Developing the usability testing protocol. Usability Testing for Survey Research.

[67] Ghasemi A, Zahediasl S (2012). Normality tests for statistical analysis: a guide for non-statisticians. Int J Endocrinol Metab.

[68] Braun V, Clarke V (2006). Using thematic analysis in psychology. Qual Res Psychol.

[69] Harris PA, Taylor R, Thielke R, Payne J, Gonzalez N, Conde JG (2009). Research electronic data capture (REDCap)--a metadata-driven methodology and workflow process for providing translational research informatics support. J Biomed Inform.

[70] Jaspers MW (2009). A comparison of usability methods for testing interactive health technologies: methodological aspects and empirical evidence. Int J Med Inform.

[71] Virzi RA (2016). Refining the test phase of usability evaluation: how many subjects is enough?. Hum Factors.

[72] Nielsen J, Landauer TK (1993). Mathematical model of the finding of usability problems. Proceedings of the INTERACT '93 and CHI '93 Conference on Human Factors in Computing Systems.

[73] Brooke J, Jordan PW, Thomas B, McClelland IL, Weerdmeester B (1996). SUS: a 'quick and dirty' usability scale. Usability Evaluation In Industry.

[74] Schnall R, Cho H, Liu J (2018). Health information technology usability evaluation scale (Health-ITUES) for usability assessment of mobile health technology: validation study. JMIR Mhealth Uhealth.

[75] Yen PY, Bakken S (2012). Review of health information technology usability study methodologies. J Am Med Inform Assoc.

[76] O'Connell S, Palmer R, Withers K, Saha N, Puntoni S, Carolan-Rees G, PROMs‚ PREMsEffectiveness Programme (2018). Requirements for the collection of electronic PROMS either "in clinic" or "at home" as part of the PROMs, PREMs and Effectiveness Programme (PPEP) in Wales: a feasibility study using a generic PROM tool. Pilot Feasibility Stud.

[77] Palmen LN, Schrier JC, Scholten R, Jansen JH, Koëter S (2016). Is it too early to move to full electronic PROM data collection?: A randomized controlled trial comparing PROM's after hallux valgus captured by e-mail, traditional mail and telephone. Foot Ankle Surg.

[78] Kyte D, Ives J, Draper H, Calvert M (2016). Current practices in patient-reported outcome (PRO) data collection in clinical trials: a cross-sectional survey of UK trial staff and management. BMJ Open.

[79] Liu TC, Ohueri CW, Schryver EM, Bozic KJ, Koenig KM (2018). Patient-identified barriers and facilitators to pre-visit patient-reported outcomes measures completion in patients with hip and knee pain. J Arthroplasty.

[80] (2020). AOA PROMs pilot project final report. Australian Orthopaedic Association National Joint Replacement Registry (AOANJRR).

[81] Lungu DA, Pennucci F, De Rosis S, Romano G, Melfi F (2020). Implementing successful systematic patient reported outcome and experience measures (PROMs and PREMs) in robotic oncological surgery-the role of physicians. Int J Health Plann Manage.

[82] Arner M (2016). Developing a national quality registry for hand surgery: challenges and opportunities. EFORT Open Rev.

[83] Morris ME, Brusco N, Woods J, Myles PS, Hodge A, Jones C, Lloyd D, Rovtar V, Clifford A, Atkinson V (2021). Protocol for implementation of the 'AusPROM' recommendations for elective surgery patients: a mixed-methods cohort study. BMJ Open.

[84] Abbasi F, Khajouei R, Mirzaee M (2020). The efficiency and effectiveness of surgery information systems in Iran. BMC Med Inform Decis Mak.

[85] Szajna B (1996). Empirical evaluation of the revised technology acceptance model. Manag Sci.

[86] Kalankesh LR, Nasiry Z, Fein RA, Damanabi S (2020). Factors influencing user satisfaction with information systems: a systematic review. Galen Med J.

[87] Aggelidis VP, Chatzoglou PD (2012). Hospital information systems: measuring end user computing satisfaction (EUCS). J Biomed Inform.

[88] Mustafa M, Alzubi S, Chakraborty C, Banerjee A, Garg L, Rodrigues JJ (2020). Factors affecting the success of internet of things for enhancing quality and efficiency implementation in hospitals sector in Jordan during the crises of COVID-19. Internet of Medical Things for Smart Healthcare: COVID-19 Pandemic.

[89] Handayani PW, Hidayanto AN, Budi I (2018). User acceptance factors of hospital information systems and related technologies: systematic review. Inform Health Soc Care.

[90] Sidani S, Epstein DR, Bootzin RR, Moritz P, Miranda J (2009). Assessment of preferences for treatment: validation of a measure. Res Nurs Health.

[91] Ahmed S, Zidarov D, Eilayyan O, Visca R (2021). Prospective application of implementation science theories and frameworks to inform use of PROMs in routine clinical care within an integrated pain network. Qual Life Res.

[92] Meirte J, Hellemans N, Anthonissen M, Denteneer L, Maertens K, Moortgat P, van Daele U (2020). Benefits and disadvantages of electronic patient-reported outcome measures: systematic review. JMIR Perioper Med.

[93] Schamber EM, Takemoto SK, Chenok KE, Bozic KJ (2013). Barriers to completion of patient reported outcome measures. J Arthroplasty.

[94] Jordan JE, Buchbinder R, Briggs AM, Elsworth GR, Busija L, Batterham R, Osborne RH (2013). The health literacy management scale (HeLMS): a measure of an individual's capacity to seek, understand and use health information within the healthcare setting. Patient Educ Couns.

[95] Knight J, Ayyash K, Colling K, Dhesi J, Ewan V, Danjoux G, Kothmann E, Mill A, Taylor S, Yates D, Ayyash R (2020). A cohort study investigating the relationship between patient reported outcome measures and pre-operative frailty in patients with operable, non-palliative colorectal cancer. BMC Geriatr.

[96] Mason JD, Blencowe NS, McNair AG, Stevens DJ, Avery KN, Pullyblank AM, Blazeby JM (2015). Investigating the collection and assessment of patient-reported outcome data amongst unplanned surgical hospital admissions: a feasibility study. Pilot Feasibility Stud.

[97] Kwong E, Black N (2018). Feasibility of collecting retrospective patient reported outcome measures (PROMs) in emergency hospital admissions. J Patient Rep Outcomes.

[98] Iversen HH, Holmboe O, Bjertnaes O (2020). Patient-reported experiences with general practitioners: a randomised study of mail and web-based approaches following a national survey. BMJ Open.

[99] Bliddal S, Banasik K, Pedersen OB, Nissen J, Cantwell L, Schwinn M, Tulstrup M, Westergaard D, Ullum H, Brunak S, Tommerup N, Feenstra B, Geller F, Ostrowski SR, Grønbæk K, Nielsen CH, Nielsen SD, Feldt-Rasmussen U (2021). Acute and persistent symptoms in non-hospitalized PCR-confirmed COVID-19 patients. Sci Rep.

[100] Wintner LM, Giesinger JM, Zabernigg A, Rumpold G, Sztankay M, Oberguggenberger AS, Gamper EM, Holzner B (2015). Evaluation of electronic patient-reported outcome assessment with cancer patients in the hospital and at home. BMC Med Inform Decis Mak.

[101] Aiyegbusi OL, Nair D, Peipert JD, Schick-Makaroff K, Mucsi I (2021). A narrative review of current evidence supporting the implementation of electronic patient-reported outcome measures in the management of chronic diseases. Ther Adv Chronic Dis.

[102] Hutchings A, Neuburger J, Grosse Frie K, Black N, van der Meulen J (2012). Factors associated with non-response in routine use of patient reported outcome measures after elective surgery in England. Health Qual Life Outcomes.

[103] Bevans M, Ross A, Cella D (2014). Patient-reported outcomes measurement information system (PROMIS): efficient, standardized tools to measure self-reported health and quality of life. Nurs Outlook.

[104] Wild D, Grove A, Martin M, Eremenco S, McElroy S, Verjee-Lorenz A, Erikson P, ISPOR Task Force for TranslationCultural Adaptation (2005). Principles of good practice for the translation and cultural adaptation process for patient-reported outcomes (PRO) measures: report of the ISPOR task force for translation and cultural adaptation. Value Health.

[105] Rao JK, Anderson LA, Inui TS, Frankel RM (2007). Communication interventions make a difference in conversations between physicians and patients: a systematic review of the evidence. Med Care.

[106] Ivers N, Jamtvedt G, Flottorp S, Young JM, Odgaard-Jensen J, French SD, O'Brien MA, Johansen M, Grimshaw J, Oxman AD (2012). Audit and feedback: effects on professional practice and healthcare outcomes. Cochrane Database Syst Rev.

[107] Pattinson RC, Say L, Makin JD, Bastos MH (2005). Critical incident audit and feedback to improve perinatal and maternal mortality and morbidity. Cochrane Database Syst Rev.

[108] de Jong RK, Snoek H, Staal WG, Klip H (2019). The effect of patients' feedback on treatment outcome in a child and adolescent psychiatric sample: a randomized controlled trial. Eur Child Adolesc Psychiatry.

[109] Skivington K, Matthews L, Simpson SA, Craig P, Baird J, Blazeby JM, Boyd KA, Craig N, French DP, McIntosh E, Petticrew M, Rycroft-Malone J, White M, Moore L (2021). A new framework for developing and evaluating complex interventions: update of Medical Research Council guidance. BMJ.

