# Supplementary material for: Usability of an Automated System for Real-Time Monitoring of Shared Decision-Making for Surgery: Mixed Methods Evaluation
Source: JMIR Hum Factors. 2024 Apr 10;11:e46698. doi: 10.2196/46698 (PMC11043934; doi:10.2196/46698)
Supplement: Multimedia Appendix 1 [file humanfactors_v11i1e46698_app1.pdf]

Multimedia Appendix 1: Example screenshots of the measurement system

1. Survey invitations

Screenshot of short messaging service invitation	Screenshot of email invitation
Dear [redacted]. You are booked for surgery at NBT. Help us to support you by completing a short survey https://sms-uk.cemplicity.com/?s=VWSTAF85 Thanks, NBT.	NHS<br>North Bristol<br>NHS Trust<br><br>Dear [redacted],<br>You've been booked for surgery at North Bristol NHS Trust. Please help us to support you by completing this short survey.<br><br>The survey will take no longer than 5 minutes to complete and it will help us improve both your future care, and the care of other patients having surgery.<br>Click here to begin<br>If you are interrupted, you can click on this link again and it will take you back to where you were up to.<br>Thank you for your help.<br><br>Yours Sincerely,<br>[redacted]<br>**Deputy Medical Director**<br><br>If you would like to read our Privacy Notice or to contact our designated Data Protection Representatives (DPR), please click here.

2. Example screenshots of survey displayed on a smartphone

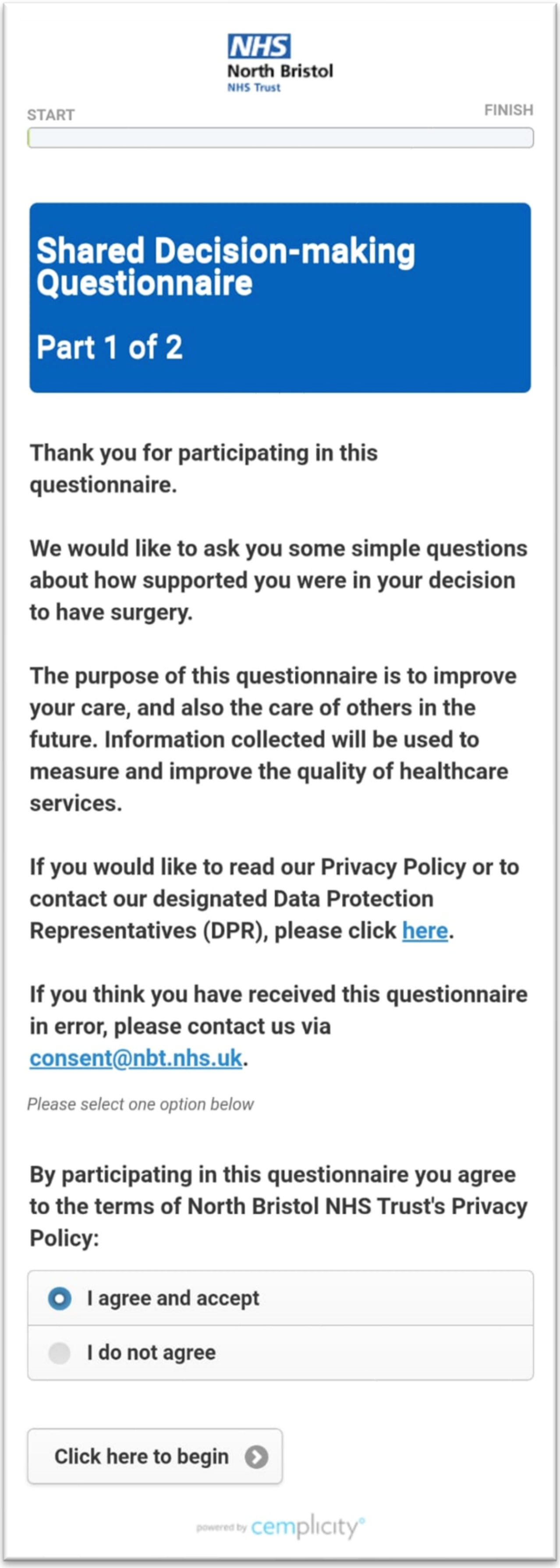

NHS
North Bristol
NHS Trust

START FINISH

Shared Decision-making Questionnaire

Part 1 of 2

Thank you for participating in this questionnaire.

We would like to ask you some simple questions about how supported you were in your decision to have surgery.

The purpose of this questionnaire is to improve your care, and also the care of others in the future. Information collected will be used to measure and improve the quality of healthcare services.

If you would like to read our Privacy Policy or to contact our designated Data Protection Representatives (DPR), please click here.

If you think you have received this questionnaire in error, please contact us via consent@nbt.nhs.uk.

Please select one option below

By participating in this questionnaire you agree to the terms of North Bristol NHS Trust's Privacy Policy:

- I agree and accept
- I do not agree

Click here to begin

powered by cemplicity

NHS
North Bristol
NHS Trust

START FINISH

Thinking about your planned operation...

How much effort was made to help you understand your health issues?

-- Please select from the list below --

How much effort was made to listen to the things that matter most to you about your health issues?

-- Please select from the list below --

How much effort was made to include what matters most to you in choosing what to do next?

-- Please select from the list below --

Back Next

powered by cemplicity

3. Example screenshots of survey displayed on an internet browser

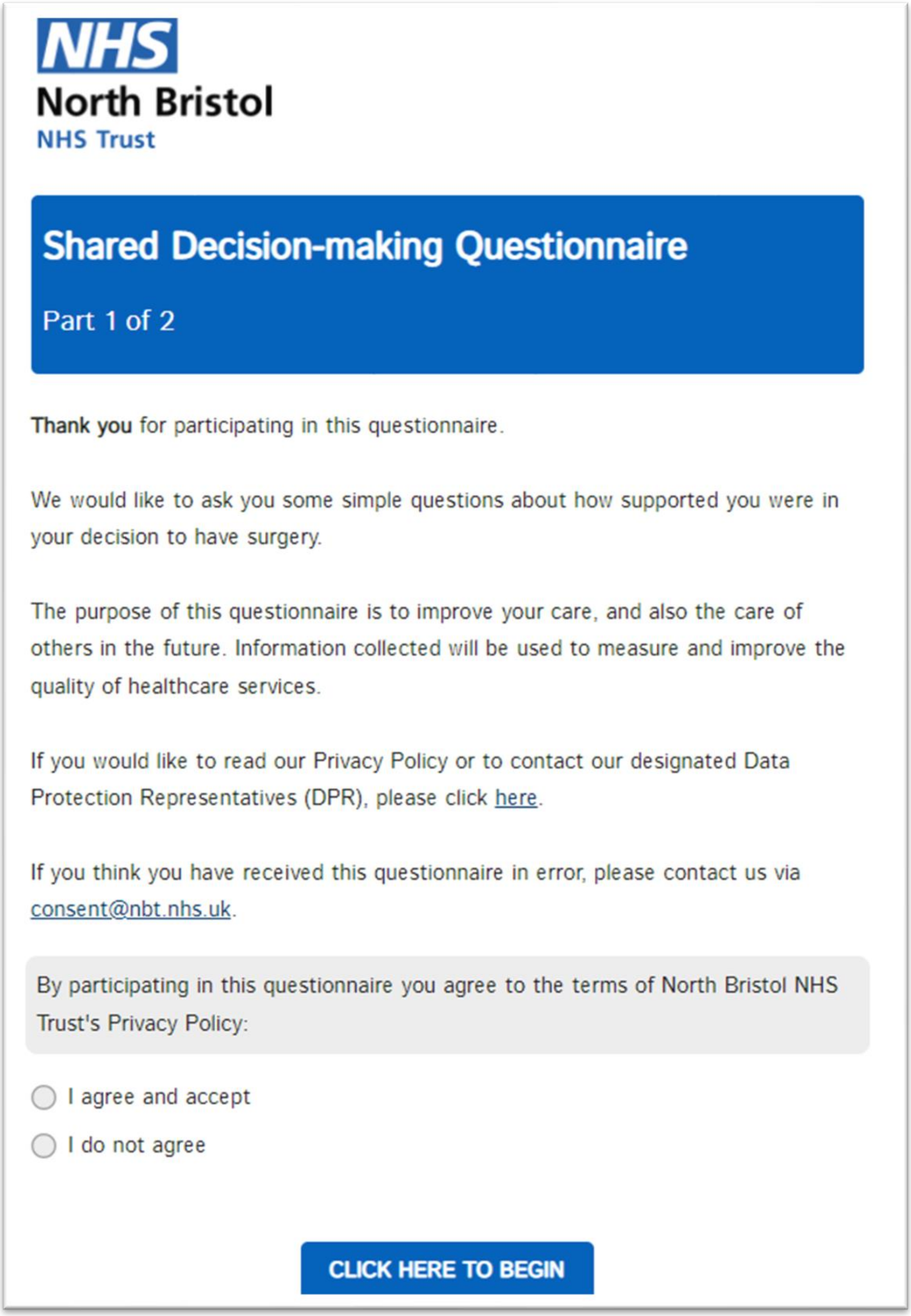

NHS
North Bristol
NHS Trust

Shared Decision-making Questionnaire

Part 1 of 2

Thank you for participating in this questionnaire.

We would like to ask you some simple questions about how supported you were in your decision to have surgery.

The purpose of this questionnaire is to improve your care, and also the care of others in the future. Information collected will be used to measure and improve the quality of healthcare services.

If you would like to read our Privacy Policy or to contact our designated Data Protection Representatives (DPR), please click here.

If you think you have received this questionnaire in error, please contact us via consent@nbt.nhs.uk.

By participating in this questionnaire you agree to the terms of North Bristol NHS Trust's Privacy Policy:

- ◯ I agree and accept
- ◯ I do not agree

CLICK HERE TO BEGIN

NHS
North Bristol
NHS Trust

Thinking about your planned operation...

How much effort was made to help you understand your health issues?

	0	1	2	3	4	5	6	7	8	9	
No effort was made	○	○	○	○	○	○	○	○	○	○	Every effort was made

How much effort was made to listen to the things that matter most to you about your health issues?

	0	1	2	3	4	5	6	7	8	9	
No effort was made	○	○	○	○	○	○	○	○	○	○	Every effort was made

How much effort was made to include what matters most to you in choosing what to do next?

	0	1	2	3	4	5	6	7	8	9	
No effort was made	○	○	○	○	○	○	○	○	○	○	Every effort was made

<< BACK <   > NEXT >>
