# Supplementary material for: Usability of an Automated System for Real-Time Monitoring of Shared Decision-Making for Surgery: Mixed Methods Evaluation
Source: JMIR Hum Factors. 2024 Apr 10;11:e46698. doi: 10.2196/46698 (PMC11043934; doi:10.2196/46698)
Supplement: Multimedia Appendix 2 [file humanfactors_v11i1e46698_app2.pdf]

Multimedia Appendix 2: Example topic guide

1. Example topic guide for usability testing sessions

Intro

- Thank you and introduction (explain project, permission to audio record, questions)
- Ask for preferred mode for completing the survey (sms/email) and send the demo link

Explain current session

- Purpose of the session:
    - We would like to test the survey that patients receive when booked in for surgery.
    - The focus is on functionality. It helps us make improvements to the process.
    - This session is NOT about the wording of questions, we are just interested in the usability
    - The text/email is a tester only, so the responses given are not real
- Specific tasks:
    - There are two surveys. 9 steps (3 questions) for the first one, 20 steps (9 questions) for the second one.
    - We will
        - run through these steps and see how you get on with these
        - might feel a little unnatural but is important you tell me what you think and what you see, what is clear/unclear, what is easy/not straight forward or difficult to complete
    - Say where there is a problem, e.g. that you had to press twice to proceed
- Reminder of questions at the end

Think-aloud exercise

Start with 9 steps of CollaboRATE

- Prompts if participant doesn't talk
    - Can you tell me what you currently see?
    - What are you going to do next?
    - What can you see now?
- Prompts to elicit views
    - Could you tell me what you think about Step X?
    - How do you feel about Step X?
    - What do you think about Step X?
    - How clear is Step X?
    - How easy is Step X?

Pause and ask follow-up questions

- "Having just completed the survey…"
    - How easy do you think is it to respond to the survey?
    - What do you think about the length of the survey?
    - What are your thoughts on the overall visual display? How visually appealing is the survey?
    - What would stop people from doing the survey? *Why?*
    - What issues can you think of people might encounter when completing the survey? *Why?*
    - What else would you change about how the survey is delivered? *Why?*

2. Example topic guide for semi-structured interviews

Intro

- Thank you and introduction (explain project, interviewee and role, reminder of anonymity, permission to audio record, questions)
- Take questions
- Assess how much of the survey completion the participant remembers

"I'm interested in your thoughts about the survey you completed. So thinking back to when you filled in the questions…"

1. What were your initial thoughts when receiving the survey? *Can you explain why you thought/felt XYZ?*

Effectiveness

2. How easy do you think was it to respond to the survey? *Why*? *What are your thoughts on the navigation, user-friendliness, interface?*

Efficiency

3. What do you think of the length of the survey? *Why*? *How quick was it to respond?*

Satisfaction with visuals

4. What were your thoughts on the visual display of the text/email?
5. And how good was the visual display of the survey itself?

Likelihood of using the system again

6. How likely do you think it is that you respond to the same survey again if there was a follow-up questionnaire?
