# Supplementary figures and images for: Usability of an Automated System for Real-Time Monitoring of Shared Decision-Making for Surgery: Mixed Methods Evaluation
Source: JMIR Hum Factors. 2024 Apr 10;11:e46698. doi: 10.2196/46698 (PMC11043934; doi:10.2196/46698)

### Multimedia Appendix 3: process map of tasks

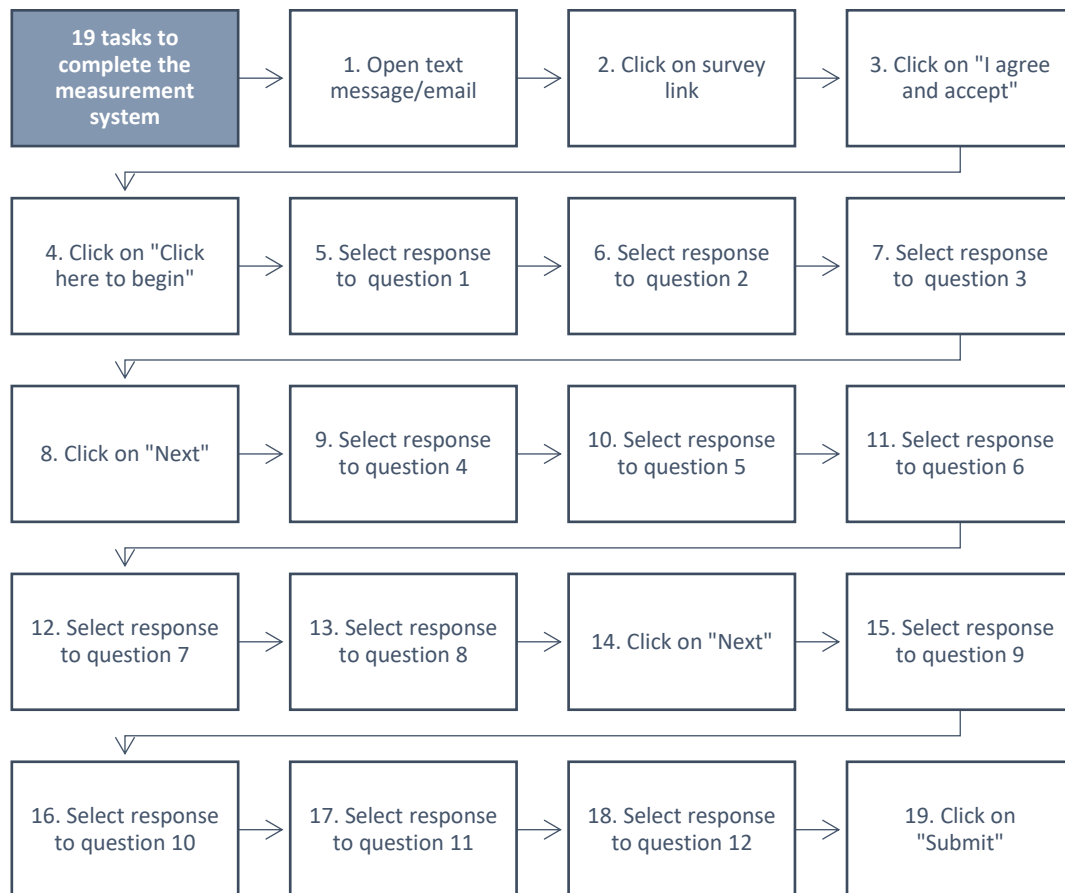

Supplement: Multimedia Appendix 3 [file humanfactors_v11i1e46698_app3.pdf]
